# Path Dependency in the Discounting of Delayed and Probabilistic Gains and Losses

**DOI:** 10.1038/s41598-019-45376-9

**Published:** 2019-06-19

**Authors:** Wojciech Białaszek, Przemysław Marcowski

**Affiliations:** Institute of Cognitive and Behavioral Neuroscience, SWPS University of Social Sciences and Humanities, Warsaw, 03-815 Poland

**Keywords:** Human behaviour, Operant learning

## Abstract

Human decision making often involves outcomes that are both risky and delayed. In such delayed lottery scenarios, the question of how such prospects are evaluated arises. An individual can arrive at their choice by following three different subjective value elicitation paths: (1) a direct path by considering the delay and risk of an outcome simultaneously; (2) a delay-probability path by first considering the delay and then the risk of an outcome; and (3) a probability-delay path by first considering the probability and then the delay of an outcome. Using a discounting framework, we conducted an experiment to investigate whether individual choices are path dependent, i.e., if the three paths elicit different subjective values of risky and delayed gains or losses. The experiment included an arbitrary selection of delays and individual probability estimates corresponding to each delay, obtained in an additional delay-probability trade-off task. Such approach ensured the equal individual decision factor strength of each outcome delay and probability. Our findings demonstrate that the human choice of risky and delayed gains or losses is indeed path dependent, which contrasts with the normative view. Furthermore, we present evidence that human choice more closely follows the delay-probability elicitation path than the probability-delay path in the domain of gains.

## Introduction

Time and risk are some of the most widely studied factors in decision-making. The majority of research (with some notable exceptions) focuses on the impact of one isolated factor on choice behavior. However, often decisions in real life are not made solely based on the delay or probability of their consequences, but their combination^1,2^. Here we report the findings from an experiment that focuses on choice scenarios that we refer to as delayed lotteries. In such scenarios, the occurrence of a rewarding or aversive outcome is linked to both the delay and probability of its receipt.

**Figure 1:**
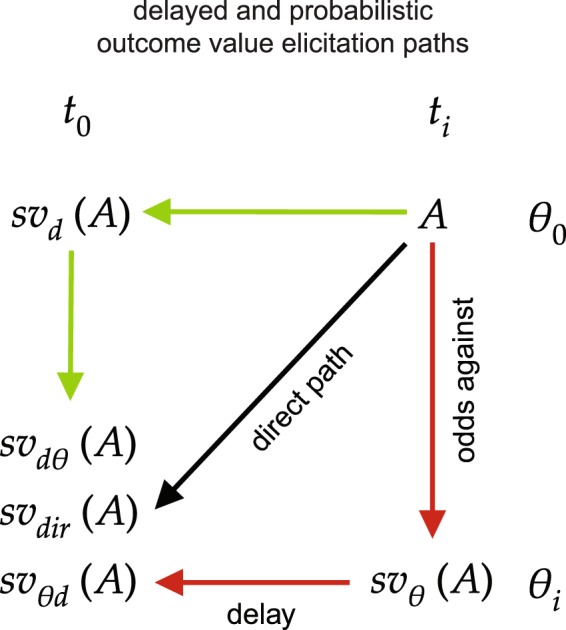
Theoretical model for delayed and risky outcome subjective value elicitation paths. Diagram representing each outcome value elicitation path that was translated into the experimental design. The initial undiscounted value of an outcome (*A*) can be first discounted by its delay and then by the probability of its receipt (expressed as odds against), yielding *sv*_dθ_(*A*). This delay-probability elicitation path is depicted in green. In the probability-delay path (red), outcome *A* is first discounted by the probability of its receipt and then by its delay. In the direct path (black), the subjective value of an outcome *A* is discounted simultaneously by the delay and probability and yields *sv*_dir_(*A*).

In choices involving delayed lotteries, when outcome *A* is both delayed and probabilistic, individuals may evaluate the outcome in three different ways or *paths*^3,4^ (Fig. 1). The first is a direct evaluation, which corresponds to estimating the immediate certainty equivalent of the delayed lottery. We refer to this scenario as a *direct* (dir) *path*, where *sv*_dir_(A) (subjective value of outcome *A*) is elicited by a direct evaluation of outcome *A* with a given delay and given probability. The second path of eliciting an immediate certainty equivalent of a delayed lottery would require a two-stage process. The first stage would be an estimation of the certainty equivalent of a delayed lottery (as though it was only risky), followed by a further estimation of the immediacy equivalent, ultimately yielding a subjective value of the complete delayed lottery. In this path, the outcome is first discounted by the probability and then by the delay, yielding *sv*_θd_(A), which we refer to as the *probability-delay* (θd) *path*. The third path would first elicit a delay-discounted value, yielding an immediacy equivalent, which is then further discounted by the risk component of a delayed lottery, resulting in *sv*_dθ_(A). We refer to this path as the *delay-probability* (dθ) *path*. We refer to probability as the odds against outcome receipt, i.e., $\theta =(1-p)/p$, where *p* refers to the outcome probability^5^.

Normatively, from the perspective of expected utility theory^6^, the three subjective value elicitation paths outlined above should yield equal values, i.e., *sv*_dir_(A) ≡ *sv*_θd_(A) ≡ *sv*_dθ_(A). However, previous studies suggest that delaying the risk reduces the aversion toward it, thus providing indirect support for path dependency. For example, Stevenson^7^ observed a seemingly counterintuitive result that equivalent monetary amounts were discounted more as a function of delay when outcome receipt was riskless compared to a choice scenario with the prospects being both delayed and risky. Stevenson hypothesized that delay might be less aversive in delayed lotteries than in standalone delayed, but certain payoffs, and that an addition of a lottery component distracts individuals from the delay in the outcome receipt. It is possible, that when delayed, not only the outcome but also risk is discounted^8^. Correspondingly, the findings that risk tolerance increases with the delay in the resolution of that risk seem quite robust, and manifest themselves in different response collection strategies, such as rating scales^9^ or commonly used risk assessment tasks^10,11^. The abovementioned studies suggest that *sv*_dir_(A) should be greater than the subjective values obtained in the two indirect paths.

To the best of our knowledge, only two previous studies have directly investigated path dependency in human decision making. The first study investigated whether the probability-delay path and a direct method of evaluating delayed lotteries yield equal results^3^. Using a matching task (in which participants directly provide a subjective value), the authors reported that the subjective values in the direct evaluation of delayed lotteries were higher than those obtained in the indirect probability-delay path. The effect generally held in both domains, but was more pronounced in the loss condition. In other words, the individuals in the matching procedure treated outcome probabilities as though they were larger than in scenarios in which the probabilities were resolved immediately.

The second study was performed by Öncüler & Onay^4^, who employed a matching procedure to investigate path dependency in the domain of hypothetical monetary gains. They found that the subjective values were higher in the direct path of pricing a delayed lottery than in the probability-delay path. Moreover, the values obtained in the delay-probability path were higher than those in the probability-delay path but not different from those in the direct path. The findings reported by the authors provide considerable support for path dependency, particularly *sv*_θd_(A) < *sv*_dθ_(A) ≈ *sv*_dir_(A).

In studies involving risky outcomes (within a discounting framework), researchers usually aim to cover a wide range of probabilities, from high through moderate to low. Doing so is quite easy to accomplish due to the finite range defined by certainty and implausibility. Conversely, the delay in intertemporal choice is only lower bound by immediacy and lacks an upper bound, i.e., outcomes can be infinitely delayed. This aspect does not present any problems when the discounting process is investigated in isolation. However, it may be problematic in investigations involving combinations of outcome delays and probabilities. For example, Weatherly *et al*.^12^ found that probability discounting alters delay discounting but that delay discounting does not alter probability discounting. In other words, the delay discounting rate varied as a function of probability, but the probability discounting rate seemed to be resistant to differences in delay. This effect might be due to the fact that arbitrarily selected delays might have lesser (and arguably difficult to compare) strength than the wide range of probabilities used in a given experiment. Therefore, the findings suggesting probability to have a more substantial role than delay in delayed lottery choice scenarios can be due to the arbitrary choice of outcome delays and probabilities.

The present research aims to determine (1) whether there is path dependency in eliciting the subjective value of delayed and risky gains and losses and (2) the extent to which delay and probability impact the resulting subjective value of gains or losses that are both delayed and probabilistic. In the reported experiment, we investigate path dependency in gains or losses using individually obtained delay-probability trade-offs and extended—compared to previous studies—delay ranges. This treatment aims to address the limitations of prior studies, which use only specific arbitrary delay and probability values, by equalizing the impact of delay and probability on the outcome value in delayed lottery scenarios. To the best of our knowledge, we report the first experiment to directly test path dependency in the evaluation of risky and delayed gains or losses using a choice procedure.

## Results

**Figure 2:**
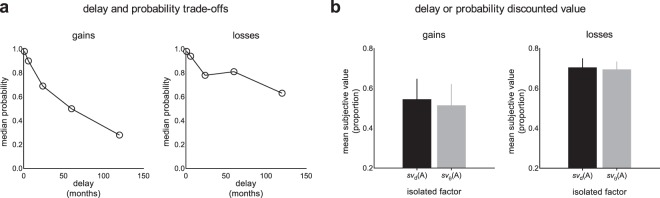
Delay and probability trade-offs and isolated effects of delay or probability. (**a**) median individual probability estimates (expressed as probability, p[0, 1]), corresponding to each arbitrary delay in the outcome receipt in the domain of gains or losses. (**b**) value elicited only by outcome delay (*sv*_d_(A)) or probability (*sv*_θ_(A)), averaged across all delays or probabilities in the domain of gains or losses. Mean subjective value is expressed as a proportion of the nominal amount, i.e., PLN 1500. The error bars represent standard errors of the mean.

During first stage of the reported experiment, we used a trade-off procedure to equalize the discounting strength of delay and probability. Essentially, this procedure aimed to find the magnitude of risk each participant was willing to accept to obtain an immediate reward or a loss (PLN 1500, equaling approximately EUR 350 at the time) instead of waiting for a time corresponding to each delay to obtain it. As can be seen in Fig. 2a, participants were less tolerant of risk with increasing delays. Upon visual inspection, individual probability estimates (corresponding to each delay) are evidently higher in the domain of losses than in the domain of gains (Fig. 2a), i.e., participants were more risk-seeking in the domain of losses than in the domain of gains (Fig. 2a). Two repeated-measures ANOVAs were performed to test whether the isolated effects of delay or probability on the subjective value of payoffs differ separately in the domain of gains or losses. In gains, there was a main effect of the indifference points (*F*(3, 262) = 279.25; *p < *0.001; η_p_^2^ = 0.736) but not of the discounting factor type (*F*(1, 100) = 3.06; *p = *0.084; η_p_^2^ = 0.030). There was no interaction between the indifference point values and the discounting factor type (*F*(3, 349) = 0.84; *p = *0.490; η_p_^2^ = 0.008). Similarly, in losses, there was a main effect of the indifference points (*F*(2, 234) = 36.78; *p < *0.001; η_p_^2^ = 0.267) but not of the discounting type (*F*(1, 101) = 0.19; *p = *0.668; η_p_^2^ = 0.002) or the interaction of the indifference points and cost type (*F*(3, 344) = 0.55; *p = *0.548; η_p_^2^ = 0.007). These analyses suggest that the delay and probability trade-off procedure was effective and equalized the impact of the probability factor to the impact of the delay factor (Fig. 2b). For example, in the longest delay of 10 years, on a group level, receiving PLN 1500 after 120 months was equally attractive as receiving PLN 1500 immediately with 28% chance.

**Figure 3:**
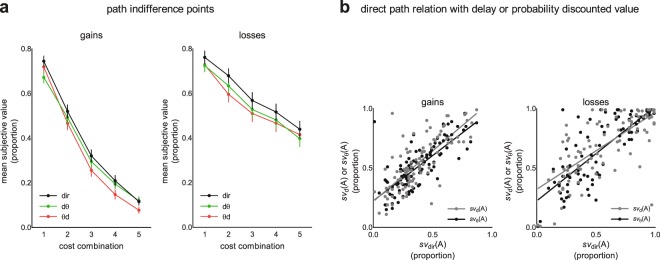
Presentation of experimental results. (**a**) mean indifference points obtained in the three outcome subjective value elicitation paths (dθ, θd, or dir) in the domain of gains or losses. Three discounting factor paths included probability, then delay discounting (θd); delay, then probability discounting (dθ); and simultaneous delay and probability discounting (dir). Proportion refers to subjective value divided by the nominal value. The OX values [1.. 5] refer to conditions with delays (shortest to longest) combined with corresponding calibrated probabilities. Mean subjective value is expressed as a proportion of the nominal amount, i.e., PLN 1500. The error bars represent standard errors of the mean. (**b**) correlation plots for the subjective values obtained in the direct path (*sv*_dir_(A)) and the values elicited only by outcome delay (*sv*_d_(A) or probability (*sv*_θ_(A)).

Two-way repeated-measures ANOVAs were then performed to compare the indifference point values obtained in the three discounting paths for all payoff delays and probabilities with fixed payoff delay times and individual probability estimates (obtained from the calibration procedure), corresponding to each delay time. In gains, there was a main effect of the elicitation path (*F*(2, 200) = 10.61; *p < *0.001; η_p_^2^ = 0.096) and of the indifference points (*F*(3, 258) = 166.68; *p < *0.001; η_p_^2^ = 0.727). The interaction of the elicitation path and indifference points was also significant (*F*(6, 628) = 2.91; *p = *0.007; η_p_^2^ = 0.028). To disentangle this interaction, we analyzed the pattern of multiple comparisons between each subjective value elicited by different elicitation paths in each indifference point (Fig. 3a). In the combination with the shortest delay and corresponding odds against, the values obtained from the direct path were higher than those obtained from delay-probability (*p* = 0.009). In the combination with the second shortest delay and corresponding odds against, no differences were significant (all *p* > 0.05); however, in the last three indifference points, there were consistent significant differences between the direct and probability-delay paths (respective *p* values: *p* < 0.001, *p* = 0.002, *p* = 0.002) and between the probability-delay and delay-probability paths (respective *p* values: *p* = 0.023, *p* < 0.001, *p* < 0.001). None of the remaining comparisons reached significance (all *p* > 0.05). This result suggests that, at least in the last three indifference points, the outcome values were greater if obtained in the direct path or the delay-probability path, compared to their equivalents in the probability-delay path.

In the domain of losses, the results reflect those in the domain of gains but without interaction. We observed a main effect of the elicitation path (*F*(2, 190) = 5.96; *p = *0.004; η_p_^2^ = 0.056) and the indifference points (*F*(2, 232) = 51.38; *p < *0.001; η_p_^2^ = 0.337) but not of the interaction of the elicitation path and indifference points (*F*(7, 661) = 0.81; *p = *0.696; η_p_^2^ = 0.007). Following post-hoc analyses showed that the indifference point values obtained in the direct path (889.52 ± 442.26, mean ± sd) were higher than those obtained in the probability-delay path (814.75 ± 474.13, mean ± sd; *p = *0.006) and in the delay-probability path (829.50 ± 449.49, mean ± sd; *p = *0.038); the differences between the indirect paths did not reach significance (*p = *0.466; Fig. 3a).

Finally, to determine the relationship between the subjective values obtained across the indifference points in the direct path and those discounted by delay or probability in isolation, we performed multiple regression analysis. In the domain of gains, after equalizing the impact of delay and probability in the trade-off task, we observed that both predictors together explained 70.0% of the variance (*F*(2, 98) = 115.71; *p* < 0.001) and that delay-discounted values significantly predicted the values obtained in the direct path (β = 0.45; *p* < 0.001), as did probability-discounted values (β = 0.47; *p* < 0.001). Following the same analytical approach, RWA (Relative Weights Analysis) showed that the RWs for delay (RW = 0.35; 95% BCa (0.22; 0.47)) and for probability (RW = 0.36; 95% BCa (0.15; 0.48)) explained a significant portion of the model variance (95% BCa (0.22; 0.46) and 95% BCa (0.13; 0.48), respectively), but their weights were not significantly different from one another because the 95% BCa test of significance included zero (−0.28; 0.23). These analyses suggest that both predictors explained a comparable portion of the variance in the direct evaluation of delayed lotteries (Fig. 3b).

Similarly, in losses, multiple regression analysis again showed that the values in the direct path were predicted by delay or probability, explaining 76.4% of the variance (*F*(2, 99) = 164.03; *p* < 0.001). Delay-discounted values successfully predicted the values obtained in the direct path (β = 0.35; *p* < 0.001), as did probability-discounted values (β = 0.61; *p* < 0.001; Fig. 3b). RWA showed that the RWs for delay (RW = 0.31; 95% BCa (0.17; 0.44) and for probability (RW = 0.46; 95% BCa (0.36; 0.57) explained a significant portion of the model variance (95% BCa (0.18; 0.39) and 95% BCa (0.36; 0.57), respectively), but their weights were not significantly different from one another because the 95% BCa test of significance included zero (−0.22; 0.38).

## General Discussion

In our experiment we demonstrated that the evaluation of delayed and risky gains or losses is indeed path dependent. Our main finding suggests that the direct path elicits greater subjective value of delayed lotteries than an indirect path, where value is elicited first by outcome probability and then by outcome delay. Conclusions as to whether the values elicited in the direct or delay-probability path differ remain to be further investigated. However, we provide support that the indirect delay-probability path yields estimates that are closer to the direct path than the probability-delay path in the domain of gains, but not in losses. In addition, we investigated the contribution of outcome delay and probability to the value estimations of delayed lotteries. We found that in gains or losses, the value of delayed lotteries is shaped by both discounting factors; however, their choice should be guided by their impact on the subjective value, not by arbitrary choices.

Delayed and risky outcomes seem to be discounted less steeply when their subjective value is elicited through the direct path than in a two-stage elicitation, i.e., evaluation first by outcome probability and then by its delay. Noussair & Wu^10^ reported that approximately 39% of participants exhibited greater risk aversion in choice scenarios with risky gains that are immediate compared to scenarios with risky gains that are additionally delayed by three months. The authors proposed that with increasing delay, the risk aversion declines. This proposition was further tested and confirmed by Abdellaoui *et al*.^13^, who showed that when lotteries were immediately resolved, 77% of participants exhibited risk aversion. However, when the delay of the lottery was increased to half a year and one year, this percentage declined to 75% and 67%, respectively (c.f. Keren & Roelofsma^14^). Relatively little is known about decision making with choice scenarios involving delayed and risky losses; however, Ahlbrecht & Weber^3^ showed that when risky losses are delayed, their aversiveness might increase, possibly due to an overestimation of probability. Similarly, delayed and probabilistic gains are perceived as more attractive when their subjective value is elicited through the direct path compared to the indirect probability-delay path. A similar mechanism may govern decision making in losses than in gains, which—if delayed—are discounted less by risk (gains are more attractive, losses are more aversive) with increasing delays.

Our findings show that in the gains condition, there were differences between the subjective values elicited in the two indirect paths: we observed a trend of *sv*_dθ_(A) being greater than *sv*_θd_(A). Generally, in gains, the two indirect paths elicited different values, but no such differences were observed in the losses condition. One possible explanation might be linked to the well-established magnitude effect observed in the discounting of delayed or probabilistic gains or losses. Delayed gains are discounted in an amount-dependent fashion–small monetary gains are discounted more steeply than large monetary gains. This phenomenon is referred to as the magnitude (or amount) effect^15–17^. In other words, delay discounts larger gains proportionally less than smaller gains. This amount-dependent discounting rate is also present in probability discounting; however, the effect is reversed–as a function of risk, small gains are discounted less steeply than large gains^18,19^. This amount-dependency is not present in losses, where both the discounting delay and probability rates are amount-insensitive^20^.

Questions remain as to why in gains—but not in losses—the two indirect elicitation paths yield different value estimates. The results from our study provide one answer. If the delay and probability trade-off task equalizes the discounting impact of delays and probabilities, then, in answering this question, it is crucial to focus on what occurs in the second elicitation stage of the indirect path, i.e., probability discounting in the delay-probability and delay discounting in the probability-delay path. The implication is that in this second elicitation stage of the indirect paths, either probability is less aversive or delay is more aversive compared to the first stage of value elicitation. The main difference is the amount of a gain or a loss in the second stage of eliciting the final subjective value, compared to the first stage. As mentioned above, the outcome magnitude in delay and probability discounting affects the discounting rate in gains but not in losses. Therefore, it could be expected that the difference in the two indirect paths should be present only in the domain of gains, but not in losses. This hypothesis is confirmed by our findings—in the gains condition, *sv*_dθ_(A) was greater *sv*_θd_(A). In both indirect paths, the nominal amount (PLN 1500) is first discounted by either delay or probability. Then, the amount which would be further discounted by the remaining discounting factor is lower. Because the amount is lower than previously, it is discounted less steeply (as predicted by the reverse magnitude effect) by probability in the delay-probability path. In the probability-delay path, where the nominal payoff is discounted first by probability, the resulting value is further discounted even more steeply by delay (compared to how a higher nominal payoff would be delay discounted). No such relationship was observed in the domain of losses due to the lack of magnitude and reverse magnitude effects present in this domain. Accordingly, in losses, *sv*_θd_(A) did not differ from *sv*_dθ_(A).

Interestingly, we also observed an interaction of the indifference points in different paths in the gains condition. In the shortest delay (1 month) and highest calibrated probability of obtaining a gain, the subjective value corresponding to that indifference point was lower in the delay-probability path than that obtained in the direct path. However, in the three longest delays, we observed consistent differences. No theoretical support for this finding is currently available. Given that no differences would be observed in the two shortest delays, this observation could thus be attributed to relatively low discounting—and therefore weaker magnitude and reverse magnitude effects. Possibly, path-dependency is only present in longer delays and not in the highest probabilities. In other words, the longer the delay and the lower the corresponding calibrated probability (higher odds against), the larger the gap between the direct path and the indirect paths—at least in the domain of gains.

Overall, our findings demonstrate path dependency in risky intertemporal choice and are supported by prior reports^3,4^. However, we also note some interesting differences. First, Ahlbrecht & Weber^3^ reported differences in the discounting rates between the direct and probability-delay paths obtained with a matching procedure, but they did not replicate these findings with a choice procedure. Indeed, several studies have concluded that there are differences in matching and choice procedures, suggesting a lack of procedure invariance^21–23^. As suggested by Tversky *et al*.^24^, different attributes of decision scenarios might carry different weights, depending on the specific elicitation procedure. Following this, Hardisty *et al*.^25^ suggested that in choice procedures with participants usually facing choices between given options, more attention may be paid to the discounting factor (i.e., delay or probability) of the larger outcome option. In matching procedures where participants are asked to note an immediacy or certainty equivalent of a delayed or probabilistic outcome, it is plausible that one might focus more on the payoff magnitude. This proposal might hold important explanatory power for the nonuniformity in our findings. It may be the case that in a matching procedure, the differences across different elicitation paths might be more pronounced than those obtained in a choice procedure due to a greater attentional focus on the outcome magnitude.

Although we did not collect data regarding the specific cognitive strategies used by the participants to arrive at their choices, our results provide some insights for further research on this topic. It seems plausible that if people follow a sequential strategy in evaluating risky future prospects, then they might first evaluate them by the associated delay and then by risk. In one of their studies, Öncüler & Onay^4^ focused on the process aspect of a matching task that involved delayed lotteries. They found that individuals used three types of information when evaluating delayed lotteries. The primary type was the information regarding the payoff value, followed by its delay, and finally the information regarding its probability. Such a finding, combined with the tendency of delay-probability path subjective values to be closer to the direct path than those in the probability-delay path, supports the conclusion that when evaluating a delayed and risky prospect, individuals first discount by delay and then by probability.

Path dependency has both theoretical and practical implications. In the field of cognitive modeling, we provide testable predictions of the possible processes in the evaluation of delayed and risky outcomes. In practical settings, our findings may be applied in financial and health decision making. For example, if an investor is offered a deal that involves a delayed and probabilistic loss, they might be more inclined to accept that deal if delay and probability is presented in succession, compared to an equivalent deal but with decision factors presented upfront. When applied to individual health decision strategies, particularly in more steeply-discounting individuals, switching the strategy (path) may support more adaptive behaviors. For example, substance use and abuse may lead to harmful consequences that are both delayed and probabilistic. Considering both delay and risk simultaneously, may be more deterrent than considering those two attributes stepwise (e.g., “smoking is bad, but the effects won’t show for a long time—and even then, I might get lucky”). This seems particularly important because reports demonstrate delay and probability discounting that discriminate among various populations, such as those exhibiting substance use and abuse^26^. Because different paths yield different consequences, it is possible that different groups and individuals may vary in the path they use in the evaluation of delayed lotteries. Further research may address if different populations vary in evaluating delayed lotteries with respect to different elicitation paths, or if path dependency is present in non-monetary outcomes, such as positive (gains) or negative (losses) health consequences. Finally, if path choice proves susceptible to experimental manipulation, then decision making might be improved by arranging situations so as to encourage use of the most effective path.

Importantly, in the present research, we observed that delays and probabilities should be selected carefully when constructing delayed lottery choice scenarios. For example, it was observed that the delay discounting rates decreased with lower probabilities of outcomes but that the probability discounting rates were generally unaffected by the delay component^12^. One possible interpretation of this observation is that the delay values used were too small for the corresponding probabilities. For example, Weatherly *et al*.^12^ included a wide range of probabilities from 0.95 to 0.01, but the longest delay of 5 years was at most moderate compared to risk. Similar ranges were used by Vanderveldt *et al*.^27^ (see also Cox & Dallery^28^), who concluded that probability might be more salient in decisions that involve delayed lotteries. Considering prior research that investigated delay and probability trade-offs^29^, the range of outcome probabilities, compared to a relatively narrow range of delays, might not be appropriate, and therefore, it results in an overall stronger impact of the probabilities. The range of probabilities is bound by certainty and implausibility; however, the range of delays is only lower bound by immediacy, which may therefore produce some artifacts. In our experiment we observed that after equalizing the impact of delays and probabilities, the weights of probability and delay discounting in the final delay lottery value estimates were also equalized.

## Methods

### Participants

The participants in the experiment were students recruited from the university student pool, and they were awarded course credit for participation. All participants signed an informed consent form prior to participating in the experiment. All experimental procedures were approved by the local ethics committee (Faculty of Psychology, SWPS University of Social Sciences and Humanities), and the experiment was performed in accordance with the relevant guidelines and regulations. A total of 203 individuals participated in the experiment (25.5 ± 7.3, mean ± sd; 72 females and 29 males in the gains condition and 82 females and 20 males in the losses condition). The experimental procedures were computer assisted and performed individually at the university behavioral laboratory.

### Procedure

**Figure 4:**
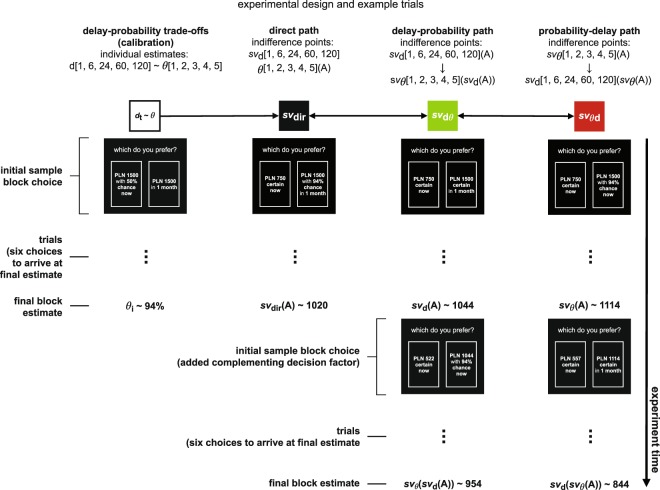
General experimental design and example trials in each outcome subjective value elicitation path. Experimental design with the three outcome value elicitation paths: the probability-delay path (θd); the delay-probability path (dθ), and the simultaneous delay and probability path (dir). Included were five different outcome delays (1, 6, 24, 60, and 120 months) and five individual delay-probability trade-offs (θ_i1_, θ_i2_, θ_i3,_ θ_i4,_ θ_i5_). Delay and probability calibration task was used to obtain individual probability trade-offs for each delay. The presentation of each path was counterbalanced across participants, as was the exposure to the discounting factor values (outcome delays and probabilities). The only condition not counterbalanced was that the primary discounting factor always appeared before the complementary factor in the delay-probability and probability-delay paths, i.e., indifference points s*v*_d_(A) and s*v*_θ_(A) were used to obtain appropriate dependent indifference points *sv*_θ_(*sv*_d_(A)) in the delay-probability path (dθ) and *sv*_d_(*sv*_θ_(A)) in the probability-delay path (θd), respectively. Example trials were included from outcomes in the domain of gains, in condition where delay was set to one month.

The experimental procedure employed the algorithm presented in Fig. 4, and was programmed to obtain a set of indifference points corresponding to the three outcome value elicitation paths, i.e., the probability-delay path (θd), the delay-probability path (dθ), and the direct path (dir) (Fig. 4). Each indifference point reflected a subjective value of a delayed, probabilistic, or delayed and probabilistic outcome. To obtain such an estimation, we employed a dynamic staircase procedure with an adjusting (decreasing or increasing) immediate value. This widely used procedure^29–31^ dynamically selects choice options based on the previous choices by a participant. The participants were presented with a series of choices between an adjusting immediate and certain outcome option and a delayed, probabilistic, or delayed and probabilistic option. Each choice was made by indicating one of two panels (left or right) presented on a computer screen, with a randomized presentation of the adjusting option on either the left or right panel.

In the reported experiment, the arbitrary outcome delays in the gains or losses conditions were 1, 6, 24, 60, and 120 months, and the complementing probabilities in each path were individually obtained in a delay and probability trade-off task presented prior to the main procedure. In addition to the 5 indifference points obtained in the trade-off task (one for each delay), a total of 25 indifference points were obtained in the main procedure: 10 discounted first by delay or probability (individually obtained); 10 were the values previously discounted by delay or probability discounted further by the complementing discounting factor (delay-probability and probability-delay paths); and 5 discounted simultaneously by delay and probability (individually obtained; direct path). The exposure to θd, dθ, and direct (dir) paths was counterbalanced across participants, as was the order of presentation of the discounting factor values (outcome delay times and probabilities). Only conditions that were not counterbalanced and appeared before subsequent measurement of further indifference points are the conditions used to obtain appropriate values that were then used to dynamically construct the decision cost combinations. Individual probability estimates corresponding to each delay time were used, obtained by calibrating probabilities to arbitrary delays through the trade-off task introduced prior to the main procedure.

In each path in the gains or losses condition, the initial nominal value of the delayed, probabilistic, or delayed and probabilistic option was set to PLN 1500, and the initial value of the adjusting option was always half of the nominal amount, i.e., PLN 750. In the gains condition, if a participant chose an immediate and certain option, then its value decreased in the following choice (making it less attractive) and increased if a larger outcome was chosen (making the immediate and certain option more attractive). In the losses condition, the adjusting value increased when chosen (making it more aversive) and decreased when the larger loss was preferred. Each indifference point was estimated with six choices in total, with the adjustment value diminishing with each successive choice according to the following formula: PLN 1500/2^*i*+*1*^, where *i* is the numerical order of that choice from choice 1 through choice *i* (in the case of choice 6, *i* = 6). The adjusting value resulting from the final choice constituted a given indifference point.

In the direct path, each indifference point was obtained by presenting the participants with choices between an immediate option and an option that was simultaneously delayed and probabilistic. The two indirect paths utilized a two-stage approach for each indifference point. The first stage consisted of presenting an immediate and certain option and an option that was only delayed (yielding *sv*_d_(A)) or probabilistic (yielding *sv*_θ_(A)), and it aimed to obtain indifference points for each delay time or probability. In the second stage, the values of each indifference point obtained in the first stage were taken and further discounted by the complementing discounting factor, i.e., the participants were presented with choices between an immediate and certain option and a probabilistic (delay-probability path) or delayed option (probability-delay path), with the subjective values of the probabilistic or delayed options taken from a given indifference point obtained in the first stage. This treatment resulted in a set of indifference points that were discounted simultaneously by outcome delay and probability (direct path; yielding *sv*_dir_(A)) or first discounted by a given delay and then a given probability (delay-probability path; yielding *sv*_dθ_(A)) or by a given probability and then a given delay (probability-delay path; yielding *sv*_θd_(A)). For example, in the direct path, a participant was asked to choose between PLN 750, obtained immediately and for certain, and PLN 1500, which was delayed by 1 month with a 70% chance of occurrence (the order of presenting delay and probability within the direct path was counterbalanced).

### Delay and probability trade-off task

The calibration procedure, i.e., the delay and probability trade-off task, was introduced before the main procedure and followed a logic similar to the core staircase algorithm. However, instead of the outcome value, the outcome probability (expressed as the percentage chance of occurrence) was adjusted trial by trial. A similar treatment was used in a prior study by Białaszek *et al*.^29^. This procedure aimed to obtain an estimate of probability equivalent to a given delay (trade-off); therefore, in successive choices, the probability of the risky option was adjusted (increased or decreased, based on participant choices), and the outcome value was kept constant. For example, the participants were initially presented with a choice between receiving or losing PLN 1500 with a chance of 50% and receiving or losing PLN 1500 with a given delay. In the gains condition, if the probabilistic option was selected, then in the next choice, the probability of receiving the risky option was decreased, or increased if previously the delayed option was chosen. In the losses condition, the procedure was analogous to that in the gains condition and differed only in that if the probabilistic option was chosen, its value then increased in the next choice, and decreased if the delayed option was preferred. The adjustment value followed the same algorithm as that described in the main staircase procedure and was set to a maximum of six choices per delay.

### Data analysis

For the main analyses, we utilized a factorial repeated-measures ANOVA. When interpreting the simple effects, we used post-hoc tests with Holm’s correction. Linear regression was performed to assess the delay and probability discounting impact on the value of delayed lotteries. To determine if one predictor was stronger than the other, RWAs were performed subsequently to linear regression. The RW represents an additive decomposition of the total model R^2^ that can be attributed to each predictor. To compare the RWs of the two predictors in the model, we utilized the approach outlined by Tonidandel & LeBreton^32^ and calculated the RWs for the two predictors. In further comparisons, we relied on bootstrapped confidence intervals (*n* = 10,000 resamples, bias-corrected and accelerated bootstrap intervals, BCa). Dataset generated for analytical procedures in the current paper is available in supplementary information section (Dataset 1) or in the Open Science Framework repository (data availability section).

## Supplementary information

Dataset 1

## Data Availability

Dataset generated for analytical procedures in the current paper is available in the Open Science Framework repository: https://osf.io/ru6n5/.
